# Exploring Indicators of Subcutaneous Tissue Fluid Accumulation in Breast Cancer-Related Lymphedema Patients Using Fractal Analysis with Virtual Volume

**DOI:** 10.1089/lrb.2022.0062

**Published:** 2023-10-17

**Authors:** Shiori Niwa, Fumiya Hisano, Ayana Mawaki, Keisuke Nakanishi, Sachiyo Watanabe, Atsushi Fukuyama, Toyone Kikumori, Kazuhiro Shimamoto, Kuniharu Imai, Etsuko Fujimoto, Chika Oshima

**Affiliations:** ^1^ Department of Integrated Health Sciences, Nagoya University Graduate School of Medicine, Nagoya, Japan.; ^2^ Kyoto University Hospital, Kyoto, Japan.; ^3^ Department of Radiological Science, Japan Health Care College, Sapporo, Japan.; ^4^ Department of Breast and Endocrine Surgery, Nagoya University Hospital, Nagoya, Japan.; ^5^ Department of Radiological and Medical Laboratory Sciences, Nagoya University Graduate School of Medicine, Nagoya, Japan.; ^6^ Ichinomiya Kenshin College School of Nursing, Ichinomiya, Japan.; ^7^ Department of Nursing Science, Faculty of Nursing and Social Welfare, Fukui Prefectural University, Eiheiji-Town, Japan.

**Keywords:** breast cancer-related lymphedema, ultrasound, fractal analysis with virtual volume, fluid accumulation

## Abstract

**Background:**

Breast cancer treatment sometimes causes a chronic swelling of the arm called breast cancer-related lymphedema (BCRL). Its progression is believed to be irreversible and is accompanied by tissue fibrosis and lipidosis, so preventing lymphedema from progressing by appropriate intervention at the site of fluid accumulation at an early stage is crucial. The tissue structure can be evaluated in real time by ultrasonography, and this study aims at assessing the ability of fractal analysis using virtual volume in detecting fluid accumulation within BCRL subcutaneous tissue via ultrasound imaging.

**Methods and Results:**

We worked with 21 women who developed BCRL (International Society of Lymphology stage II) after unilateral breast cancer treatment. Their subcutaneous tissues were scanned with an ultrasound system (Sonosite Edge II; Sonosite, Inc., FUJIFILM) using a 6- to 15-MHz linear transducer. Then, a 3-Tesla MR system was used to confirm fluid accumulation in the corresponding area of the ultrasound system. Significant differences in both *H* + 2 and complexity were observed among the three groups (with hyperintense area, without hyperintense area, and unaffected side) (*p* < 0.05). *Post hoc* analysis (Mann–Whitney U test; Bonferroni correction *p* < 0.0167) revealed a significant difference for “complexity.” The evaluation of the distribution in Euclidean space showed that the variation of the distribution decreased in the order of unaffected, without hyperintense area, and with hyperintense area.

**Conclusion:**

The “complexity” of the fractal using virtual volume seems to be an effective indicator of the presence or absence of subcutaneous tissue fluid accumulation in BCRL.

## Introduction

Breast cancer-related lymphedema (BCRL) of the upper extremities is a major complication after breast cancer surgery. Its clinical stage is established via the International Society of Lymphology (ISL) classification (Table 1).^1^ Even though the recommended standard care for patients with BCRL is determined by the ISL classification, this classification is based solely on interview and palpation without an understanding of the internal structure of the subcutaneous tissue.

**Table 1.** Clinical Criteria of Staging Lymphedema in the Consensus Document of the International Society of Lymphology 2016

Stage	Clinical document
Stage 0	A latent or subclinical condition is not evident despite impaired lymph transport.
Stage I	Early accumulation of fluid with relatively high protein content subsides with extremity elevation. Pitting may occur.
Stage II	Extremity elevation alone rarely reduces tissue swelling, and pitting is manifested.
Later in Stage II	The limb may not pit as excess subcutaneous fat and fibrosis develop.
Stage III	Lymphostatic elephantiasis where pitting can be absent, trophic skin changes including acanthosis, further deposition of fat and fibrosis and warty overgrowths have developed.

Appropriate intervention can improve subcutaneous tissue changes in stage II, where limb elevation alone rarely reduces tissue swelling, with manifestation of pitting, as these are not yet irreversible.^1^ We reported in a previous article that mixed results (i.e., with or without fluid accumulation) could be observed on magnetic resonance imaging (MRI) after all patients classified with stage II lymphedema received similar treatment.^2^

However, as lymphedema progresses, the corresponding tissue fibrosis and lipidosis are believed to become irreversible.^1,3,4^ Hence, it is crucial to prevent lymphedema from progressing by intervening appropriately at the site of fluid accumulation at an early stage when it is recognized.

MRI is useful for observation of lymphedema fluid accumulation, but its high cost and time and physical burden impede its common use.^3,5,6^ This motivated us to focus on diagnostic ultrasound (US) equipment, which has been successfully used in studying lymphedema.^7–10^

We have previously investigated fluid accumulation of subcutaneous tissue using texture analysis to evaluate it on US images.^11^ Texture analysis is excellent for reproducibility and quantitative evaluation^12,13^; however, it is not yet fully understood how the obtained values relate to the actual visual evaluation. Therefore, this study focuses on fractal analysis using virtual volumes, which has the characteristic of delivering results similar to human visual cognition.^14^ This is an image feature that contains a composite of information about the coarseness, distribution, and complexity of the overall texture of the image.

The purpose of this study was to perform fractal analysis using this virtual volume and verify whether this analysis was effective in evaluating the fluid accumulation status of lymphedema. If the fractal analysis is found to be appropriate for the evaluation of fluid accumulation within BCRL subcutaneous tissue, the use of fractal analysis for US images can serve as a superior examination method over MRI.

## Materials and Methods

### Subject

The study cohort consisted of 21 women, aged 45–74 years (mean 56.2, standard deviation 8.5 years), treated for unilateral breast cancer at the lymphedema outpatient unit of our institution between January 2018 and July 2020, who had subsequently developed BCRL (ISL stage II). The inclusion criteria were as follows: (1) those diagnosed with BCRL (ISL stage II) by a clinician, (2) those attending an outpatient lymphedema clinic, (3) those receiving the same conservative treatment for lymphedema after mastectomy, (4) those who had completed radiation therapy and chemotherapy at least 6 months before the study, and (5) those without the progression of the primary disease, that is, without recurrence or metastasis. All subjects had undergone axillary lymph node dissection, and the mean duration of BCRL was 41.8 (standard deviation 31.3) months.

### US imaging

#### US image capture

Subcutaneous tissue was scanned through a US system (Sonosite Edge II; Sonosite, Inc., FUJIFILM) using a 6- to 15-MHz linear transducer. During the scan, anatomical posturing was used to position the subjects. The probe was laced longitudinally on the arm afterward, with the transducer head perpendicular to the skin^15,16^ at a standardized distance from it to allow capturing images with parallel epidermis and where the deep fascia is clear within the transducer.^17^ A gel pad (HydroAid 3 mm; KIKGEL, Poland) was used at the measurement site to enable clear visualization of the most superficial layer of the skin and to prevent from excessive pressure on the skin tissue during measurements and from skin thickness modifications during probe manipulation. The gain setting was consistently used to provide images, the whole cohort having the same image display condition.

As in our previous study,^11^ the elbow heads of both arms were used as landmarks. Four points were scanned: (1) 5 cm proximal outside, (2) 5 cm proximal inside, (3) 5 cm distal outside, and (4) 5 cm distal inside to the olecranon in both arms of each patient. Two images of each point on both arms were obtained by an experienced operator. The measurement dimensions were set to 4.0 cm wide and 5.0 cm deep to capture the full depth of the subcutaneous tissue. For processing and analysis, we saved the conventional US static images as JPEG files.

### US image processing

Similar to that in previous studies,^7,9,11^ the region of interest (ROI) was set in the entire subcutaneous tissue (480 pixels horizontally, arbitrary vertical direction) on each US image using ImageJ (ImageJ, FIJI) image processing software. The size of the ROI was set to enclose the entire subcutaneous tissue because this analysis method is characterized by the ability to view the entire image information within the ROI regardless of its size.^18^ The set ROI was calculated using programming software with built-in formulas.

#### Fractal analysis using virtual volume

We performed fractal analysis by using a technique that avoids the binarization process (as the binarization process is difficult to perform under uniform image processing conditions in US images) for target images called “virtual volume.”^14,18,19^

Let us briefly describe fractal analysis. An image intensity is denoted as a sampled and quantized gray level image *f (x, y)*, and the four corner points of the square box with a side length *r* in the *(x, y)* plane are *(x, y)*, *(x + r, y)*, *(x, y + r)*, and *(x + r, y + r)*.

**FIG. 1.**
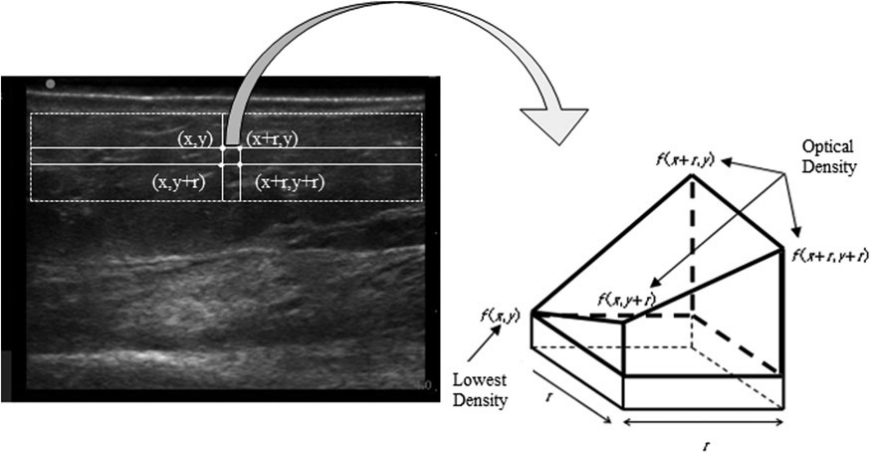
Schematic illustration of virtual volume. Four points *(x, y), (x + r, y), (x, y + r)*, and *(x + r, y + r)* are the vertices of the *square* image of ROI set throughout the subcutaneous tissue on the ultrasound image, and *f(x, y)*, *f(x + r, y)*, *f(x, y + r)*, and *f(x + r, y + r)* indicate the optical densities at these points, respectively. If optical density *f(x, y) = f0* is the lowest of them, the heights of the considered polyhedron are *f(x + r, y) − f0*, *f(x, y + r) − f0*, and *f(x + r, y + r) − f0.* ROI, region of interest.

In the three-dimensional Euclidian space (x, y, f(x, y)), when the minimum *f0* among the values *f (x, y)*, *f (x + r, y)*, *f (x, y + r)*, and *f (x + r, y + r)* is *f (x, y)*, the polyhedron with vertices *(x, y, f0)*, *(x + r, y, f0)*, *(x, y + r, f0)*, *(x + r, y + r, f0)*, *(x + r, y, f (x + r, y))*, *(x, y + r, f (x, y + r))*, and *(x + r, y + r, f (x + r, y + r))* is considered as depicted in Figure 1. More precisely, *f (x + r, y) − f0, f (x, y + r) − f0* and *f (x + r, y + r) − f0* are taken as its heights, while its volume is the already mentioned “virtual volume.”^14^ By *V(r)*, we denote the mean virtual volume across all possible ones of polyhedrons with a square base of side length *r*. The following equation is assumed as satisfied for a fractal image:

(1) V=μ

where *μ* and *a* are constants. When a coincides with *D*, it is called fractal dimension; otherwise, it is a pseudo-fractal dimension. The relationship between *V(r)* and *r* is linear on a log–log scale, namely


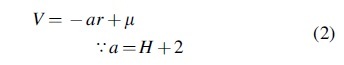


Following the fractal sequence concept, all image texture information is captured in Eq. (1). Hence, besides a pseudofractal dimension, *log_10_ μ* delivers additional relevant information; thus, it is defined as a complexity.^14,18,19^

We also consider a parameter called the Hurst index, defined by means of the Hurst index and fractal dimension as

(3) H=1−D

The complexity and the Hurst index were evaluated in this study as parameters for fractal analysis using virtual volumes.

### Magnetic resonance imaging

#### MRI sequence

We used a 3-Tesla MRI scanner (MAGNETOM Verio 3T; Siemens Healthcare Gmbh, Erlangen, Germany) to perform MRI in three-dimensional double-echo steady state (DESS) using fat suppression to observe fluid accumulation.^20^

To ease the physical burden of the patients, we shortened the acquisition time by using a type of simultaneous acquisition of spatial harmonics, and we chose the DESS imaging parameters as follows: repetition time = 14.16 ms, echo time = 5.00 ms, flip angle = 28, bandwidth = 250 Hz/pixel, field of view = 256 mm, number of slabs = 1, slices per slab = 160, and slice thickness = 1.00 mm.

Moreover, to reduce the image distortion, a two-dimensional distortion correction filter was used. We divided the area from the axilla to the wrist into three sections, namely the upper, middle, and lower regions, including the shoulder joint, elbow, and wrist, respectively. The acquisition time was 8 minutes per section (24 minutes in total). Marks were made on the skin 5 cm above and below the cubital fossa and olecranon so that the corresponding transverse MR image (MRI) could be selected.

#### Determination of the hyperintense area in the MRIs

The open-source software program Horos (Horos Project DICOM image viewing and measuring) was used for obtaining three-dimensional images from the original DICOM files. Corresponding two-dimensional MRIs with a cross section of US imaging were extracted following the marking reflected in the three-dimensional MRIs.

Two-dimensional MRI analysis was used by a panel of five experienced observers to determine the presence or absence of hyperintense areas, defined as follows: (1) a hyperintense area was noticed in the subcutaneous tissue around the virtual cross section in which the probe was placed; (2) the form of the hyperintense area did not matter (punctate, macular filamentous, and various other shapes); and (3) we assumed that there is no signal increase if the subcutaneous tissue does not exhibit one, even when the skin presents a signal increase.^11^

### Statistical analysis

SPSS (IBM SPSS Statistics version 27.0) was used for statistical analysis. We give the results as means (standard deviations). Non-normal distributions (Shapiro–Wilk test, *p* = 0.000–0.004) led to the selection of nonparametric analysis (significance set at *p* < 0.05), using the Kruskal–Wallis test to determine differences between the three groups; the Mann–Whitney U test with Bonferroni corrections (significance set at *p* < 0.0167) was used for *post hoc* analysis.

### Analysis of distribution status by hyperintense area classification

To investigate the distribution of “H+2” and the “complexity” of fractal analysis using virtual volumes for each hyperintense classification, Morisita's overlap index (a statistical measure for individuals' dispersion in a population usually used in ecology for determining sample overlaps and designed to avoid the influence of the data number per cluster; its value ranges between 0 and 1)^21,22^ was used to evaluate the distribution situation in the Euclidean space.

### Ethics

The Ethics Committee of Nagoya University (Nos. 2015–0058 and 2019-0339-4) and our research cooperation facility (No. 1323) have approved this study. Written informed consent was obtained from all participants.

## Results

### Determining the hyperintense area in the MRIs

The US images were split into three groups taking into consideration the presence of a hyperintense area (i.e., with hyperintense area, without hyperintense area, and unaffected side), in agreement with several observers. We found 38 US images with and 130 without a hyperintense area, while 168 had an unaffected side.

### Comparison between the presence of hyperintense area classification

A nonparametric Kruskal–Wallis test was used for determining possible significant differences between the three groups (with hyperintense area [*n* = 38], without hyperintense area [*n* = 130], and unaffected side [*n* = 168]) in *H* + 2 and complexity.

Table 2 shows that there are marked significant differences among the three groups in both *H* + 2 and complexity.

**Table 2.** Comparison Between the Presence of Hyperintense Area Classification

	Affected side		Unaffected side (*n* = 168)	*p*-value
	With hyperintense area (*n* = 38)	Without hyperintense area (*n* = 130)		
*H* + 2	2.506 ± 0.069	2.529 ± 0.118	2.567 ± 0.137	0.020^*^
Complexity	12.501 ± 0.207	12.640 ± 0.232	12.600 ± 0.220	0.004^*^

^*^ Kruskal–Wallis test = *p* < 0.05.

### *Post hoc* analysis between the presence of hyperintense area classification

Subsequent group comparisons were made in *H* + 2 and complexity, where significant differences were found based on the results of the Kruskal–Wallis test. For *H* + 2, no significant differences were found between any of the groups. For complexity, there were significant differences between “with hyperintense area” and “without hyperintense area” and between the first one and “unaffected side,” while there were no significant differences between “without hyperintense area” and “unaffected side” (Table 3).

**Table 3.** *Post hoc* Analysis Between the Presence of Hyperintense Area Classification

	With hyperintense area vs. without hyperintense area	With hyperintense area vs. unaffected side	Without hyperintense area vs. unaffected side
*H* + 2	0.445	0.023	0.027
Complexity	0.001^*^	0.011^*^	0.161

^*^ Mann–Whitney U test = *p* < 0.017.

### Distribution status by hyperintense area classification

**FIG. 2.**
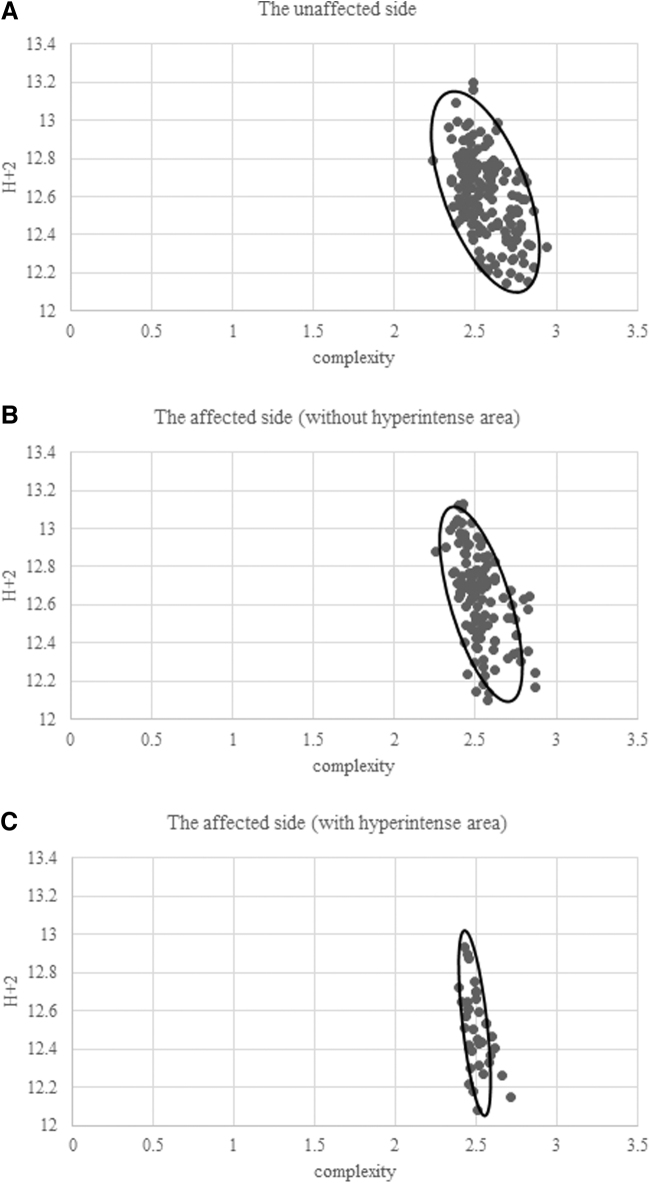
Distribution status by hyperintense area classification **(A)** the unaffected side, **(B)** the affected side (without hyperintensity area), and **(C)** the affected side (with hyperintensity area).

The distribution of the three groups mentioned above is presented in Figure 2, where one can note that these three groups were distributed in the Euclidean space in a mixed state, rather than independently of each other. However, the distribution variability became smaller and tended to converge to a certain region in the order of “unaffected side,” “without hyperintense area,” and “with hyperintense area.”

To verify these results quantitatively, we evaluated the results using Morisita's overlap index and found that the index for “with hyperintense area” was 0.1053, the one for “without hyperintense area” 0.0891, and the one for “the unaffected side” 0.0671, indicating that the variability of the distribution was smaller.

## Discussion

Hyperintense signals show in fluid-sensitive MRI sequences the presence of free water and other distinctive patterns in the affected upper limbs of BCRL patients.^5,23^ For “complexity,” there was a significant difference between “with fluid accumulation” and “without fluid accumulation” and between “with fluid accumulation” and “unaffected,” but not between “without fluid accumulation” and “unaffected.” Therefore, the “complexity” of the fractal using virtual volume is suggested to be effective in detecting the presence or absence of subcutaneous tissue fluid accumulation in BCRL.

In the evaluation of the distribution in the Euclidean space, Morisita's overlap index increased in the order of unaffected side, without fluid accumulation, and with fluid accumulation. This implies an increase in the concentration of the distribution, that is, a decrease in variability. The Morisita's overlap index for the unaffected side was 0.0671, implying that the distribution was less concentrated and the variability was high. This finding indicates that the US images on the unaffected side may have been affected by individual differences and the measurement site. On the contrary, the Morisita's overlap index of the US image with fluid accumulation on the affected side was 0.1053, indicating that the distribution was more concentrated than that on the unaffected side and showed less variability.

Thus, in US images with fluid accumulation on the affected side, water retention is a characteristic feature of the image, and the variability is considered to be low. For images without fluid retention (Morisita's overlap index: 0.0891), the variability of the image itself due to site and individual differences was reduced compared with the unaffected side when the image was changed to a lymphedema-specific image; however, detailed image characteristics were unknown in this study. Therefore, a further detailed analysis is needed. These findings suggest that fractal analysis by virtual volume, an evaluation index similar to visual cognition, can be used to evaluate fluid accumulation in lymphedema.

Essential to reliably determine the water distribution in lymphedema, MRI is impractical in daily practice settings owing to its high cost. Instead, we consider that the US system can accurately assess the current edema status in real time and can thus assist in providing the appropriate care for the condition. We are exploring quantitative measures to capture the water distribution in lymphedema using only US equipment. In a previous study, seven features of texture analysis were found to be effective in detecting fluid accumulation in lymphedema,^11^ while in the present one, significant difference in complexity were found in fractal analysis using the virtual volume technique, which showed results similar to visual perception, suggesting that it is possible to assess the distribution of water in lymphedema.

Early detection of lymphedema is essential for early and appropriate intervention at the site of fluid retention and prevention of lymphedema progression. Therefore, many lymphedema patients would benefit if measures to capture the water distribution in lymphedema using only US equipment could be implemented.

## Conclusion

In this study, we examined whether fractal analysis using virtual volume, which shows results similar to visual perception, can be used for capturing the status of fluid accumulation in the subcutaneous tissue of lymphedema. The following results were obtained.

(1) There is a real possibility of quantitatively capturing fluid accumulation in the subcutaneous tissue at the level of complexity via this technique.
(2) The distribution in the Euclidean space with *H* + 2 and complexity axes revealed that the distribution was concentrated in the order of unaffected side, affected side, and affected side (with fluid accumulation) in Morisita's overlap index.
